# 2-Bromo-1,2-diphenylethenyl 4-methyl­phenyl sulfoxide

**DOI:** 10.1107/S1600536809042184

**Published:** 2009-10-17

**Authors:** M. Krishnaiah, R. Ravi Kumar, Thanzaw Oo, Thetmar Win, S. Ghouse Peeran

**Affiliations:** a Department of Physics, S.V. University, Tirupati 517502, India; b Department of Physics, Yangon University, Myanmar; c Department of Chemistry, Sri Krishnadevaraya University, Anantapur, India

## Abstract

In the title compound, C_21_H_17_BrO_2_S, the two phenyl rings attached to the ethene group are oriented at dihedral angles of 76.19 (10) and 57.99 (8)° with respect to the Br—C=C—S plane [r.m.s. deviation 0.003 Å]. The sulfonyl-bound phenyl ring forms a dihedral angle of 83.26 (8)° with the above plane. The crystal structure is stabilized by weak C—H⋯π inter­actions.

## Related literature

For the anti­bacterial activity of sulfone compounds, see: Mandell & Sande (1985 ▶). For a related structure, see: Wolf (1999 ▶). For bond-length data, see: Allen *et al.* (1987 ▶).
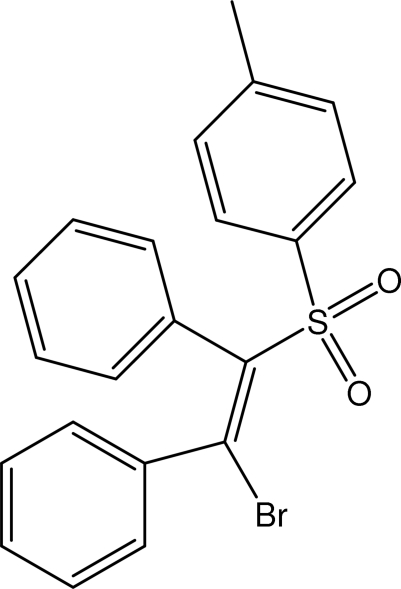


## Experimental

### 

#### Crystal data

C_21_H_17_BrO_2_S
*M* *_r_* = 413.32
Monoclinic, 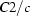

*a* = 21.561 (9) Å
*b* = 8.505 (4) Å
*c* = 21.134 (10) Å
β = 106.044 (9)°
*V* = 3725 (3) Å^3^
*Z* = 8
Mo *K*α radiation
μ = 2.33 mm^−1^
*T* = 300 K
0.15 × 0.12 × 0.08 mm

#### Data collection

Bruker SMART CCD area-detector diffractometer
Absorption correction: multi-scan (*SADABS*, Bruker, 2001 ▶) *T*_min_ = 0.661, *T*_max_ = 0.820
12736 measured reflections
4273 independent reflections
2826 reflections with *I* > 2σ(*I*)
*R*_int_ = 0.035

#### Refinement

*R*[*F* ^2^ > 2σ(*F* ^2^)] = 0.042
*wR*(*F* ^2^) = 0.109
*S* = 1.01
4273 reflections
227 parameters
H-atom parameters constrained
Δρ_max_ = 0.58 e Å^−3^
Δρ_min_ = −0.30 e Å^−3^

### 

Data collection: *SMART* (Bruker 2001 ▶); cell refinement: *SAINT* (Bruker 2002 ▶); data reduction: *SAINT*; program(s) used to solve structure: *SHELXS97* (Sheldrick, 2008 ▶); program(s) used to refine structure: *SHELXL97* (Sheldrick, 2008 ▶); molecular graphics: *PLATON* (Spek, 2009 ▶); software used to prepare material for publication: *enCIFer* (Allen *et al.*, 2004 ▶), *PARST* (Nardelli, 1995 ▶) and *PLATON*.

## Supplementary Material

Crystal structure: contains datablocks I, global. DOI: 10.1107/S1600536809042184/ci2936sup1.cif

Structure factors: contains datablocks I. DOI: 10.1107/S1600536809042184/ci2936Isup2.hkl

Additional supplementary materials:  crystallographic information; 3D view; checkCIF report

## Figures and Tables

**Table 1:** Hydrogen-bond geometry (Å, °)

*D*—H⋯*A*	*D*—H	H⋯*A*	*D*⋯*A*	*D*—H⋯*A*
C12—H12⋯*Cg*1^i^	0.93	2.91	3.608 (5)	133
C19—H19⋯*Cg*1^ii^	0.93	2.88	3.786 (4)	166

Symmetry codes: (i) 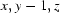; (ii) 
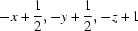
. *Cg*1 is the centroid of the C3–C8 ring.

**Fig. 1.**
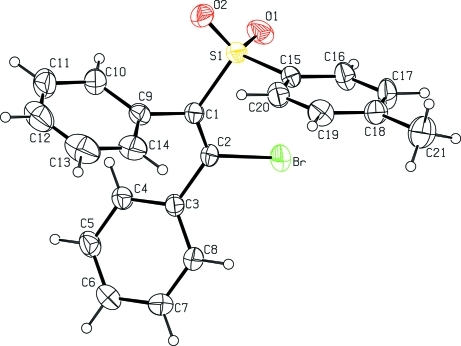
The molecular structure of the title compound, with the atom-labelling scheme. Displacement ellipsoids are drawn at the 30% probability level.

## supplementary crystallographic information

### Comment

Sulfone compounds, similar to sulfonamides, show strong *in vitro* and *in vivo* antibacterial activity, and for almost 60 years they have been used successfully in medicine (Mandell & Sande, 1985). Certain sulfones also exhibit fungicidal activity.

The separation of the Br and S1 atoms [3.371 (2) Å] is less than the sum of their respective van der Waals radii 3.65 Å. Shortening of this interatomic distance has often been observed in α,α-unsubstituted β-ketosulfones and is usually explained by hyperconjugative cross-interaction involving the π^*^(C2—Br)–σ(S1—C1) and π(C2—Br)–σ^*^(S1—C1) pairs of bonding and non-bonding molecular orbitals. According to general theory of the anomeric effect, the largest overlapping of these orbitals should occur when the interacting polar bonds are situated in the *gauche* position. However, in the title compound, the S1—C1 and C2—Br bonds are almost planar [the S1—C1—C2—Br torsion angle is 0.9 (3)°]. The only existing *gauche* interactions involve S1O1 with the C1—C2 and C1—C9 bonds. In addition, the O1···C9 non-bonding distance [3.827 (2) Å] is much longer than the sum of the respective van der Waals radii (3.22 Å). Therefore, the main electronic interaction, despite the unfavoured planar arrangement, should be the Coulombic type, weak electronic interaction of the negatively charged bromine atom and the highly positive S atom, that is responsible for the electron-density transfer from the sulfonyl group towards the bromine atom. All the above features are similar to those reported for 4'-{[benzoyl(4-tolyl-hydrazono)methyl]sulfonyl}acetanilide (Wolf, 1999, and references thererin). The bond lengths are consistent with values reported by Allen *et al.* (1987), and indicate high level of electron-density delocalization which exists in the planar phenyl rings attached to the ethene group.

### Experimental

*cis*-Stilbene (4 g) was reacted with bromine (5 g) at 283 K to obtain dibromostilbene. The resultant compound was refluxed with paramethylphenyl sodium sulphonate (4.5 g) for 6 h. The reaction mixture was condensed to yield 5 mg of the title compound and was recrystallized from methanol.

### Refinement

H-atoms were positioned at calculated positions [C—H = 0.93 Å (aromatic) and 0.96 Å (methyl)] and refined as riding with *U*_iso_(H) = 1.2*U*_eq_(C_aromatic_) and *U*_iso_(H) = 1.5*U*_eq_(C_methyl_).

### Crystal data

**Table:**

C_21_H_17_BrO_2_S	*F*(000) = 1680
*M**_r_* = 413.32	*D*_x_ = 1.474 Mg m^−^^3^*D*_m_ = 1.48 Mg m^−^^3^*D*_m_ measured by not measured
Monoclinic, *C*2/*c*	Melting point: 500 K
Hall symbol: -C 2yc	Mo *K*α radiation, λ = 0.71073 Å
*a* = 21.561 (9) Å	Cell parameters from 4223 reflections
*b* = 8.505 (4) Å	θ = 2–25°
*c* = 21.134 (10) Å	µ = 2.33 mm^−^^1^
β = 106.044 (9)°	*T* = 300 K
*V* = 3725 (3) Å^3^	Plate, colourless
*Z* = 8	0.15 × 0.12 × 0.08 mm

### Data collection

**Table:**

Bruker SMART CCD area-detector diffractometer	4273 independent reflections
Radiation source: fine-focus sealed tube	2826 reflections with *I* > 2σ(*I*)
graphite	*R*_int_ = 0.035
ω scans	θ_max_ = 28.6°, θ_min_ = 2.0°
Absorption correction: multi-scan (*SADABS*, Bruker, 2001)	*h* = −28→27
*T*_min_ = 0.661, *T*_max_ = 0.820	*k* = −11→11
12736 measured reflections	*l* = −28→28

### Refinement

**Table:**

Refinement on *F*^2^	Primary atom site location: structure-invariant direct methods
Least-squares matrix: full	Secondary atom site location: difference Fourier map
*R*[*F*^2^ > 2σ(*F*^2^)] = 0.042	Hydrogen site location: inferred from neighbouring sites
*wR*(*F*^2^) = 0.109	H-atom parameters constrained
*S* = 1.01	*w* = 1/[σ^2^(*F*_o_^2^) + (0.0592*P*)^2^] where *P* = (*F*_o_^2^ + 2*F*_c_^2^)/3
4273 reflections	(Δ/σ)_max_ = 0.008
227 parameters	Δρ_max_ = 0.58 e Å^−^^3^
0 restraints	Δρ_min_ = −0.30 e Å^−^^3^

### Special details

**Table:**

Geometry. All e.s.d.'s (except the e.s.d. in the dihedral angle between two l.s. planes) are estimated using the full covariance matrix. The cell e.s.d.'s are taken into account individually in the estimation of e.s.d.'s in distances, angles and torsion angles; correlations between e.s.d.'s in cell parameters are only used when they are defined by crystal symmetry. An approximate (isotropic) treatment of cell e.s.d.'s is used for estimating e.s.d.'s involving l.s. planes.
Refinement. Refinement of *F*^2^ against ALL reflections. The weighted *R*-factor *wR* and goodness of fit *S* are based on *F*^2^, conventional *R*-factors *R* are based on *F*, with *F* set to zero for negative *F*^2^. The threshold expression of *F*^2^ > σ(*F*^2^) is used only for calculating *R*-factors(gt) *etc*. and is not relevant to the choice of reflections for refinement. *R*-factors based on *F*^2^ are statistically about twice as large as those based on *F*, and *R*- factors based on ALL data will be even larger.

### Fractional atomic coordinates and isotropic or equivalent isotropic displacement parameters (Å^2^)

**Table:**

	*x*	*y*	*z*	*U*_iso_*/*U*_eq_	
C1	0.11848 (11)	0.3985 (3)	0.54204 (12)	0.0459 (6)	
C9	0.09957 (13)	0.2624 (3)	0.49692 (13)	0.0500 (6)	
C3	0.12159 (11)	0.5778 (3)	0.44958 (12)	0.0440 (6)	
C2	0.12993 (11)	0.5387 (3)	0.51983 (12)	0.0458 (6)	
C4	0.06193 (13)	0.5579 (3)	0.40419 (13)	0.0514 (6)	
H4	0.0273	0.5194	0.4177	0.062*	
C6	0.10397 (15)	0.6526 (4)	0.31844 (14)	0.0645 (8)	
H6	0.0981	0.6782	0.2744	0.077*	
C10	0.03670 (16)	0.2091 (4)	0.47825 (15)	0.0657 (8)	
H10	0.0056	0.2583	0.4943	0.079*	
C8	0.17237 (13)	0.6357 (3)	0.42858 (14)	0.0534 (7)	
H8	0.2126	0.6497	0.4586	0.064*	
C13	0.1272 (3)	0.0614 (5)	0.4310 (2)	0.0968 (13)	
H13	0.1579	0.0117	0.4146	0.116*	
C7	0.16352 (16)	0.6726 (4)	0.36342 (15)	0.0635 (8)	
H7	0.1979	0.7113	0.3495	0.076*	
C5	0.05398 (13)	0.5952 (4)	0.33917 (14)	0.0606 (7)	
H5	0.0139	0.5811	0.3089	0.073*	
C14	0.14498 (17)	0.1862 (4)	0.47292 (17)	0.0698 (9)	
H14	0.1877	0.2198	0.4853	0.084*	
C12	0.0654 (3)	0.0094 (5)	0.41319 (19)	0.1024 (14)	
H12	0.0542	−0.0770	0.3854	0.123*	
C11	0.0197 (2)	0.0831 (4)	0.43584 (18)	0.0896 (12)	
H11	−0.0229	0.0487	0.4228	0.108*	
S1	0.12699 (3)	0.35497 (10)	0.62701 (3)	0.0549 (2)	
O2	0.10422 (10)	0.1974 (3)	0.62829 (10)	0.0762 (7)	
O1	0.09830 (8)	0.4768 (3)	0.65579 (9)	0.0712 (6)	
C15	0.21083 (12)	0.3535 (3)	0.66457 (12)	0.0480 (6)	
C19	0.31330 (15)	0.2364 (4)	0.67791 (16)	0.0632 (8)	
H19	0.3388	0.1601	0.6659	0.076*	
C20	0.24851 (14)	0.2377 (3)	0.64798 (15)	0.0574 (7)	
H20	0.2300	0.1617	0.6169	0.069*	
C18	0.34187 (13)	0.3447 (4)	0.72521 (15)	0.0608 (7)	
C16	0.23801 (13)	0.4638 (4)	0.71088 (13)	0.0614 (7)	
H16	0.2127	0.5421	0.7219	0.074*	
C17	0.30353 (14)	0.4575 (4)	0.74120 (15)	0.0695 (8)	
H17	0.3220	0.5316	0.7732	0.083*	
C21	0.41371 (15)	0.3392 (5)	0.7578 (2)	0.0926 (12)	
H21A	0.4274	0.4369	0.7801	0.139*	
H21B	0.4362	0.3226	0.7250	0.139*	
H21C	0.4232	0.2546	0.7891	0.139*	
Br	0.158444 (16)	0.71426 (4)	0.575988 (15)	0.07199 (15)	

### Atomic displacement parameters (Å^2^)

**Table:**

	*U*^11^	*U*^22^	*U*^33^	*U*^12^	*U*^13^	*U*^23^
C1	0.0358 (12)	0.0608 (16)	0.0377 (14)	−0.0047 (11)	0.0045 (11)	0.0023 (12)
C9	0.0575 (16)	0.0513 (15)	0.0386 (15)	−0.0036 (12)	0.0089 (13)	0.0057 (11)
C3	0.0453 (14)	0.0448 (13)	0.0384 (14)	−0.0040 (11)	0.0057 (11)	−0.0029 (11)
C2	0.0351 (12)	0.0582 (16)	0.0393 (14)	−0.0076 (11)	0.0025 (11)	−0.0033 (12)
C4	0.0445 (14)	0.0641 (17)	0.0430 (15)	−0.0024 (12)	0.0077 (12)	0.0001 (13)
C6	0.076 (2)	0.0751 (19)	0.0416 (16)	0.0031 (16)	0.0147 (16)	0.0003 (14)
C10	0.070 (2)	0.0679 (19)	0.0540 (19)	−0.0146 (16)	0.0086 (16)	−0.0039 (15)
C8	0.0487 (15)	0.0611 (17)	0.0465 (16)	−0.0117 (12)	0.0067 (12)	−0.0028 (13)
C13	0.157 (4)	0.062 (2)	0.086 (3)	0.016 (2)	0.057 (3)	0.002 (2)
C7	0.0661 (19)	0.073 (2)	0.0571 (19)	−0.0133 (15)	0.0270 (16)	−0.0028 (15)
C5	0.0522 (16)	0.080 (2)	0.0426 (16)	0.0032 (14)	0.0011 (13)	−0.0013 (14)
C14	0.080 (2)	0.068 (2)	0.065 (2)	0.0041 (16)	0.0277 (18)	0.0069 (16)
C12	0.193 (5)	0.053 (2)	0.063 (2)	−0.025 (3)	0.039 (3)	−0.0082 (17)
C11	0.120 (3)	0.074 (2)	0.062 (2)	−0.040 (2)	0.004 (2)	−0.0026 (18)
S1	0.0430 (4)	0.0810 (5)	0.0379 (4)	−0.0120 (3)	0.0064 (3)	0.0061 (3)
O2	0.0724 (14)	0.0939 (17)	0.0528 (13)	−0.0361 (12)	0.0013 (11)	0.0181 (11)
O1	0.0492 (11)	0.1182 (18)	0.0501 (12)	0.0083 (11)	0.0201 (10)	−0.0003 (12)
C15	0.0441 (14)	0.0618 (16)	0.0341 (13)	−0.0061 (12)	0.0040 (11)	0.0036 (12)
C19	0.0581 (18)	0.0678 (19)	0.062 (2)	0.0121 (14)	0.0142 (16)	0.0040 (15)
C20	0.0605 (18)	0.0576 (17)	0.0497 (17)	−0.0025 (13)	0.0077 (14)	−0.0031 (13)
C18	0.0455 (15)	0.079 (2)	0.0522 (18)	−0.0030 (15)	0.0040 (13)	0.0099 (16)
C16	0.0529 (16)	0.078 (2)	0.0465 (17)	0.0060 (14)	0.0023 (13)	−0.0129 (14)
C17	0.0566 (17)	0.085 (2)	0.0548 (19)	−0.0065 (16)	−0.0054 (14)	−0.0188 (16)
C21	0.0491 (18)	0.127 (3)	0.088 (3)	0.000 (2)	−0.0033 (18)	0.009 (2)
Br	0.0854 (3)	0.0743 (2)	0.0511 (2)	−0.02668 (16)	0.01018 (17)	−0.01482 (14)

### Geometric parameters (Å, °)

**Table:**

C1—C2	1.330 (4)	C5—H5	0.93
C1—C9	1.483 (4)	C14—H14	0.93
C1—S1	1.793 (3)	C12—C11	1.362 (6)
C9—C10	1.379 (4)	C12—H12	0.93
C9—C14	1.382 (4)	C11—H11	0.93
C3—C8	1.381 (3)	S1—O1	1.426 (2)
C3—C4	1.386 (3)	S1—O2	1.430 (2)
C3—C2	1.483 (3)	S1—C15	1.762 (3)
C2—Br	1.901 (3)	C15—C16	1.365 (4)
C4—C5	1.374 (4)	C15—C20	1.382 (4)
C4—H4	0.93	C19—C20	1.365 (4)
C6—C5	1.361 (4)	C19—C18	1.373 (4)
C6—C7	1.381 (4)	C19—H19	0.93
C6—H6	0.93	C20—H20	0.93
C10—C11	1.380 (5)	C18—C17	1.368 (4)
C10—H10	0.93	C18—C21	1.512 (4)
C8—C7	1.373 (4)	C16—C17	1.382 (4)
C8—H8	0.93	C16—H16	0.93
C13—C12	1.356 (6)	C17—H17	0.93
C13—C14	1.367 (5)	C21—H21A	0.96
C13—H13	0.93	C21—H21B	0.96
C7—H7	0.93	C21—H21C	0.96
			
C2—C1—C9	121.2 (2)	C13—C12—C11	120.2 (4)
C2—C1—S1	124.1 (2)	C13—C12—H12	119.9
C9—C1—S1	114.63 (18)	C11—C12—H12	119.9
C10—C9—C14	118.7 (3)	C12—C11—C10	119.8 (4)
C10—C9—C1	120.9 (3)	C12—C11—H11	120.1
C14—C9—C1	120.3 (3)	C10—C11—H11	120.1
C8—C3—C4	119.2 (2)	O1—S1—O2	118.75 (14)
C8—C3—C2	120.9 (2)	O1—S1—C15	108.95 (13)
C4—C3—C2	119.9 (2)	O2—S1—C15	107.45 (14)
C1—C2—C3	124.9 (2)	O1—S1—C1	109.93 (13)
C1—C2—Br	122.9 (2)	O2—S1—C1	105.77 (12)
C3—C2—Br	112.19 (18)	C15—S1—C1	105.13 (11)
C5—C4—C3	120.0 (2)	C16—C15—C20	120.4 (2)
C5—C4—H4	120.0	C16—C15—S1	120.1 (2)
C3—C4—H4	120.0	C20—C15—S1	119.5 (2)
C5—C6—C7	119.4 (3)	C20—C19—C18	121.8 (3)
C5—C6—H6	120.3	C20—C19—H19	119.1
C7—C6—H6	120.3	C18—C19—H19	119.1
C9—C10—C11	120.3 (3)	C19—C20—C15	119.1 (3)
C9—C10—H10	119.8	C19—C20—H20	120.5
C11—C10—H10	119.8	C15—C20—H20	120.5
C7—C8—C3	120.1 (3)	C17—C18—C19	118.0 (3)
C7—C8—H8	120.0	C17—C18—C21	121.5 (3)
C3—C8—H8	120.0	C19—C18—C21	120.5 (3)
C12—C13—C14	120.7 (4)	C15—C16—C17	119.1 (3)
C12—C13—H13	119.6	C15—C16—H16	120.4
C14—C13—H13	119.6	C17—C16—H16	120.4
C8—C7—C6	120.5 (3)	C18—C17—C16	121.5 (3)
C8—C7—H7	119.8	C18—C17—H17	119.2
C6—C7—H7	119.8	C16—C17—H17	119.2
C6—C5—C4	120.9 (3)	C18—C21—H21A	109.5
C6—C5—H5	119.5	C18—C21—H21B	109.5
C4—C5—H5	119.5	H21A—C21—H21B	109.5
C13—C14—C9	120.1 (4)	C18—C21—H21C	109.5
C13—C14—H14	119.9	H21A—C21—H21C	109.5
C9—C14—H14	119.9	H21B—C21—H21C	109.5
			
C2—C1—C9—C10	−105.1 (3)	C14—C13—C12—C11	1.3 (6)
S1—C1—C9—C10	77.0 (3)	C13—C12—C11—C10	−1.4 (6)
C2—C1—C9—C14	75.1 (3)	C9—C10—C11—C12	1.1 (5)
S1—C1—C9—C14	−102.8 (3)	C2—C1—S1—O1	45.3 (2)
C9—C1—C2—C3	3.9 (4)	C9—C1—S1—O1	−136.93 (19)
S1—C1—C2—C3	−178.41 (19)	C2—C1—S1—O2	174.6 (2)
C9—C1—C2—Br	−176.77 (18)	C9—C1—S1—O2	−7.6 (2)
S1—C1—C2—Br	0.9 (3)	C2—C1—S1—C15	−71.9 (2)
C8—C3—C2—C1	−122.7 (3)	C9—C1—S1—C15	105.9 (2)
C4—C3—C2—C1	58.0 (4)	O1—S1—C15—C16	−3.0 (3)
C8—C3—C2—Br	57.9 (3)	O2—S1—C15—C16	−132.9 (2)
C4—C3—C2—Br	−121.4 (2)	C1—S1—C15—C16	114.8 (2)
C8—C3—C4—C5	0.2 (4)	O1—S1—C15—C20	175.4 (2)
C2—C3—C4—C5	179.5 (3)	O2—S1—C15—C20	45.5 (2)
C14—C9—C10—C11	−0.7 (5)	C1—S1—C15—C20	−66.8 (2)
C1—C9—C10—C11	179.5 (3)	C18—C19—C20—C15	1.6 (5)
C4—C3—C8—C7	−0.1 (4)	C16—C15—C20—C19	−0.9 (4)
C2—C3—C8—C7	−179.4 (3)	S1—C15—C20—C19	−179.2 (2)
C3—C8—C7—C6	0.1 (5)	C20—C19—C18—C17	−1.1 (5)
C5—C6—C7—C8	−0.3 (5)	C20—C19—C18—C21	179.5 (3)
C7—C6—C5—C4	0.4 (5)	C20—C15—C16—C17	−0.4 (4)
C3—C4—C5—C6	−0.4 (4)	S1—C15—C16—C17	178.0 (2)
C12—C13—C14—C9	−0.9 (6)	C19—C18—C17—C16	−0.2 (5)
C10—C9—C14—C13	0.6 (5)	C21—C18—C17—C16	179.2 (3)
C1—C9—C14—C13	−179.6 (3)	C15—C16—C17—C18	0.9 (5)

### Hydrogen-bond geometry (Å, °)

**Table:**

*D*—H···*A*	*D*—H	H···*A*	*D*···*A*	*D*—H···*A*
C12—H12···Cg1^i^	0.93	2.91	3.608 (5)	133
C19—H19···Cg1^ii^	0.93	2.88	3.786 (4)	166

Symmetry codes: (i) *x*, *y*−1, *z*; (ii) −*x*+1/2, −*y*+1/2, −*z*+1.
